# Reflective metalens with sub-diffraction-limited and multifunctional focusing

**DOI:** 10.1038/s41598-017-13004-z

**Published:** 2017-10-03

**Authors:** Hui Yang, Guanhai Li, Xiaofang Su, Guangtao Cao, Zengyue Zhao, Xiaoshuang Chen, Wei Lu

**Affiliations:** 1 National Laboratory for Infrared Physics, Shanghai Institute of Technical Physics, Chinese Academy of Sciences, 500 Yu Tian Road, Shanghai, 200083 China; 2 University of Chinese Academy of Science, No.19A Yuquan Road, Beijing, 100049 China; 3 College of Physics, Mechanical and Electrical Engineering, Jishou University, Jishou, 416000 China

## Abstract

We propose an ultra-thin planar reflective metalens with sub-diffraction-limited and multifunctional focusing. Based on the equal optical path principle, the specific phase distributions for multifunction focusing are derived. Following the formulas, on-center focusing with the characteristics of sub-diffraction-limited, high focusing efficiency (85%) and broadband focusing is investigated in detail. To demonstrate the flexibility of the reflective metalens, off-center and dual spots focusing (at the horizontal and longitudinal directions) are demonstrated. Note that all these focusings are sub-diffraction-limited due to the evanescent-filed enhancement mechanism in our elaborately designed structure. The designed reflective metalens will find important applications in super-resolution imaging, microscopes, and spectroscopic designs.

## Introduction

Metasurfaces, two-dimensional planar variation on the concept of metamaterials, have been engineered to realize exotic electromagnetic properties, which are rarely found in nature^1^. Typically patterned with nano-antennas or apertures, metasurfaces have been verified to be an excellent candidate for manipulating light in subwavelength domain due to the ability to introduce arbitrary abrupt phase shifts. In virtue of their flexibility and effectiveness in shaping the wavefront, metasurfaces have promised a variety of applications such as anomalous refraction or reflection^1–5^, focusing^6–10^, optical vortex^1,11–13^ and so forth. Among them, focusing a light with miniature and ultrathin characteristics has attracted enormous interest for its wide-spread applications in laser-based microscopy, imaging and spectroscopy.

Optical lens, which can converge or diverge the straight beams, has been verified to be an indispensable element in optical systems. The traditional lens, capable of reshaping the wavefront of the beam via gradual phase accumulation along the optical paths, which is restricted by the thickness and refractive index of a given dielectric. Besides, it remains as a challenge to fabricate lenses with both short focal length and large numerical aperture (N.A.). By using the Fresnel lens designing method, the mass and volume of material can be reduced, but the thickness of the lens is still on the wavelength scale and the efficiency is reduced dramatically^7^.

Recently, tremendous advances have been obtained in the field of metasurfaces, which opened up a new door for building miniature planar metalens. By using the metasurface, a variety of plasmonic metalenses based on nano-antenna or nanoslit arrays have been investigated and experimentally demonstrated^7,8,13–18^. However, the high ohmic losses of plasmonic materials and fundamental limitations have prevented the realization of high efficiency metalens^6,19,20^. To solve such an issue, the dielectric metalens is widely adopted, with which we can achieve high focusing efficiency^10,21–28^. However, most of the previous reported metalenses are diffraction-limited, which arise from the loss of the fine-feature information carried by the high wave-vector evanescent waves^29–32^. The metalens with high efficiency and sub-diffraction-limited focusing is in great request for applications in super-resolution imaging and lithography.

In this paper, an ultra-thin planar reflective metalens that capable of realizing sub-diffraction-limited and multifunctional focusing is demonstrated. The unit cell of the metalens is optimized to function as a half-waveplate, with which a phase coverage of 2*π* can be achieved. Then, the phase compensation mechanism is discussed and required phase distribution for multifunctional focusing is derived in detail. Following the formulas, on-center, off-center, and dual-spots focusing (at horizontal and longitudinal directions) are exhibited. For the on-center focusing, the sub-diffraction-limited and broadband focusing characteristics are investigated in detail. Finally, the dependence of N.A. on the focusing area is discussed.

## Results

### Designs and structure

**Figure 1:**
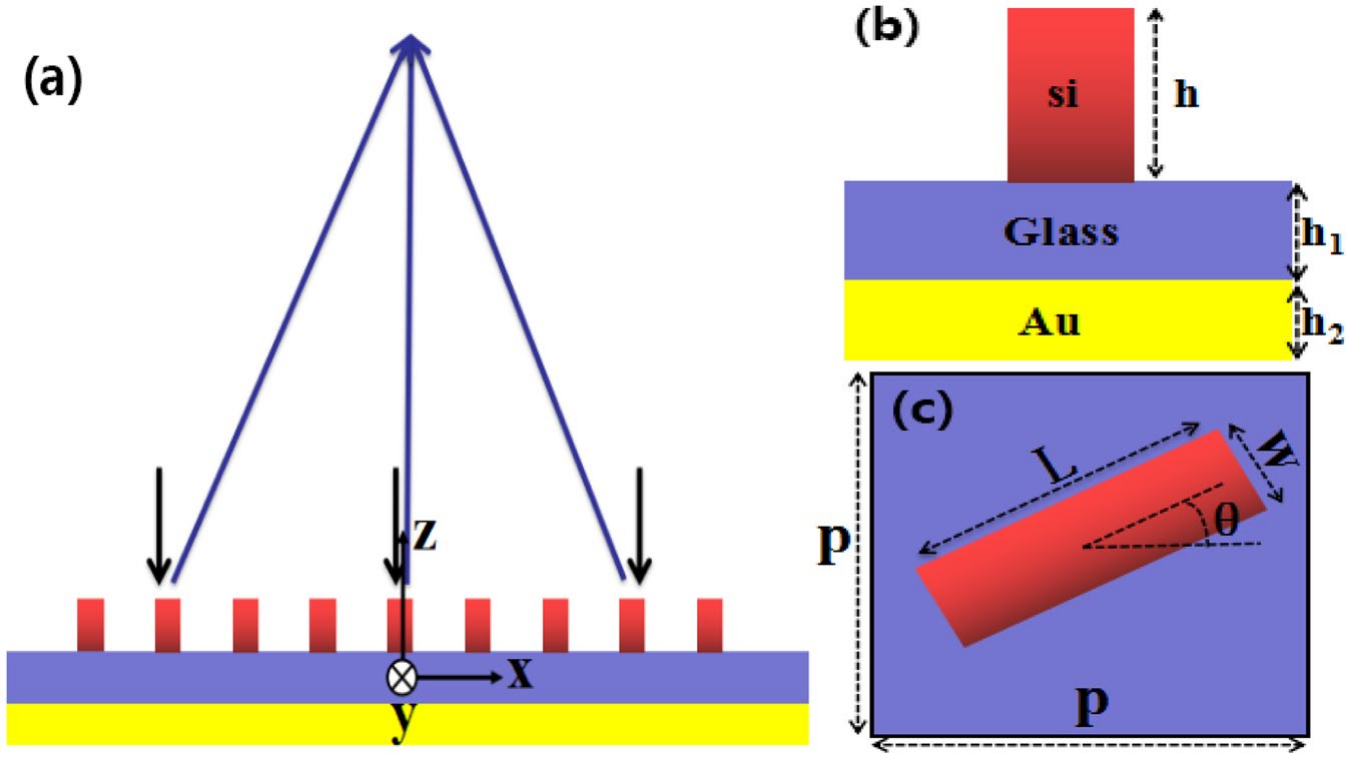
(**a**) Schematic of the reflective metalens with the black and blue arrow lines represent the incident light and reflected light, respectively. (**b**) Side view of the unit cell. The reflective metalens consists of Si nanoblocks and an Au ground plane, with a SiO_2_ spacer placed between them. The heights are *h* = 400 nm, *h*_1_ = 200 nm and *h*_2_ = 150 nm. (**c**) Top view of the unit cell with *W* = 132 nm, *L* = 600 nm and lattice constant *P* = 650 nm. The required phase is imparted by rotating the nanoblock with an angle *θ*.

Figure 1a shows the schematic of the reflective metalens. It is composed of amorphous silicon (n = 3.6) nanoblocks array and a gold ground plane with a dielectric spacer (n = 1.46) between the two layers. The side view of the unit cell is shown in Fig. 1b, with the heights from top to bottom are *h* = 400 nm *h*_1_ = 200 nm *h*_2_ = 150 nm, respectively. Top view of the unit cell is shown in Fig. 1c, from which we can see that the nanoblock’s width *W* = 132 nm, length *L* = 600 nm and the lattice constant *P* = 650 nm. The required phase can be imparted by rotating the nanoblock with an proper angle *θ*. In our proposed structure, the parameters of the nanoblocks are optimized by using the three-dimensional finite difference time domain (FDTD) method from Lumerical Inc.

To achieve a reflective metalens with high focusing efficiency, the nanoblocks should perform as a half-waveplate which is able to convert the circular polarized light into reflected light with opposite helicity. Here the nanoblock is selected for its simple structure and strong polarization conversion effect. Similar to other nanostructures with azimuthal asymmetry, nanoblock can also exhibits form birefringence^33^. A single nanoblock resembles a channel waveguide, which will have a corresponding effective refractive index (*n*_eff_) for the two orthogonal linear polarization states, *E*_x_ and *E*_y_. For the channel waveguide that owns a circular cross-section, the neff for both linear polarized light (*E*_x_ and *E*_y_) are the same. However, for our designed nanoblock that owns a rectangle cross-section, the effective refractive index neff for *E*_x_ and *E*_y_ is different, which is equivalent to form birefringence^34^. Therefore, under circularly polarized incident light, the nanoblock function as a waveplate and high polarization conversion efficiency can be achieved.

**Figure 2:**
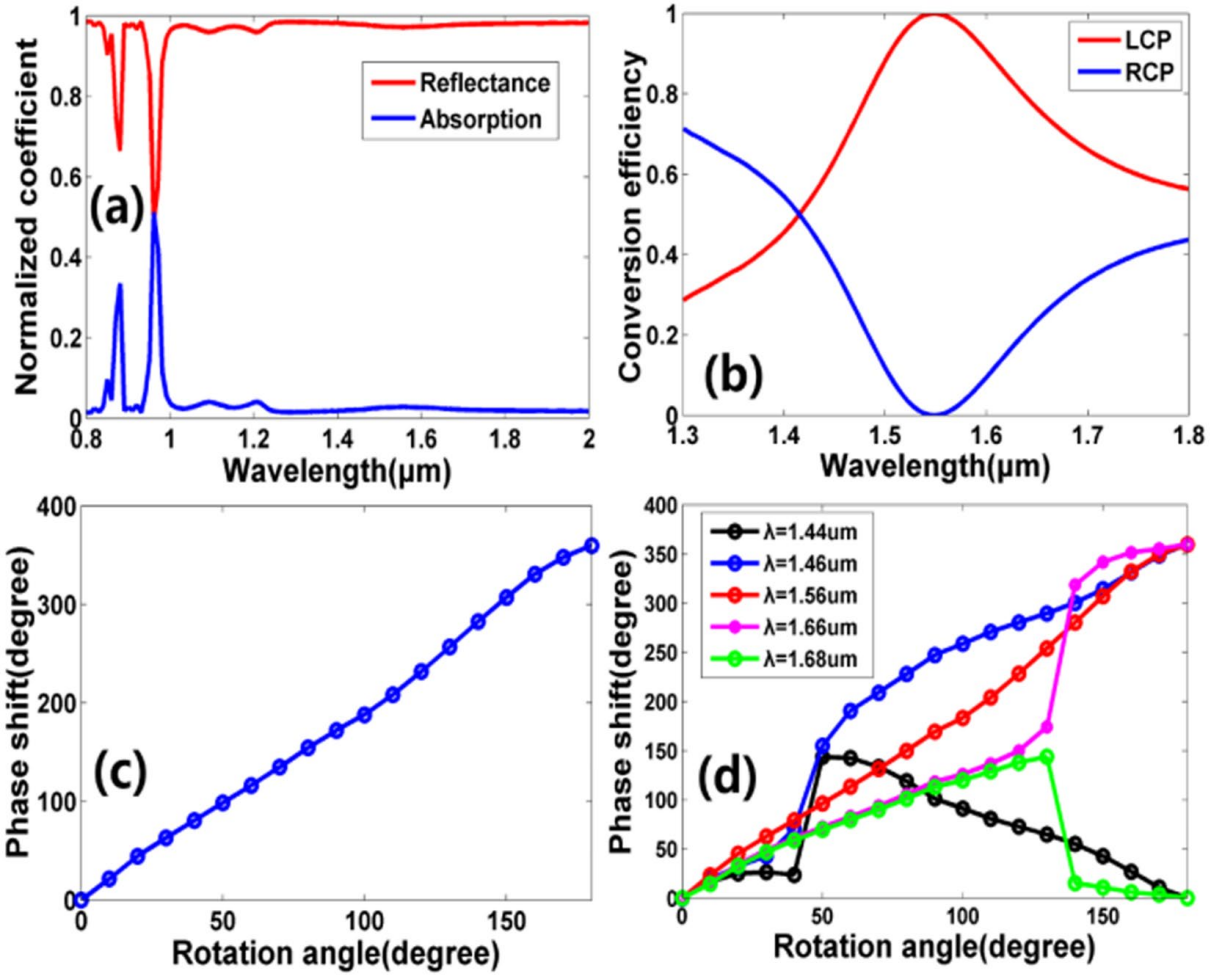
(**a**) Simulated reflection and absorption coefficient of the unit cell as a function of the incident wavelength. (**b**) Simulated polarization conversion efficiency of the unit cell as a function of the incident wavelength. The red and blue lines represent the proportion of the LCP and RCP in the total reflected power, respectively. (**c**) Simulated phase shift for the nanoblock with rotation angle *θ* ranges from 0° to 180° with a step angle of 10° at wavelength *λ* = 1550 nm. (**d**) Simulated phase shift of the nanoblock with different rotation angle *θ* for various incident wavelengths. For these simulations, periodic boundary conditions are applied along the *x* and *y* axis and perfectly matched layers (PML) is applied along the *z* axis.

Figure 2a,b shown the reflectance and conversion efficiency of the nanoblock as a function of the incidence wavelength, respectively. It can be observed that both the reflectance and conversion efficiency reach their peaks at the designed wavelength (*λ* = 1550 nm). In this case, the reflectance reaches 96% with a polarization conversion efficiency as high as 99.6%. Here, the polarization conversion efficiency is calculated as the ratio of the reflected power with opposite helicity to the total reflected power. According to the definition in ref.^27^. which is defined as the ratio of the reflected power with opposite helicity to the total incident power, the polarization conversion efficiency should be 95.6%. The phase shift of the nanoblock with various rotation angle *θ* are plotted in Fig. 2c. It can be observed that the phase shift and the rotation angle satisfy the condition of Pancharatnam-Berry (P-B) phase (*φ* = 2*θ*), from which the phase shift coverage of 0 to 2*π* is obtained^27,35^. The dependence of phase shift on the rotation angle *θ*, for various incident wavelengths, is shown in Fig. 2d. For a wavelength range of 200 nm (from 1.46 μm to 1.66 μm), the phase shift can achieve a coverage of 2*π*, indicating the broadband characteristic of our designed nonablock.

In order to focus an incident plane wave, a phase compensation mechanism is required at the surface of metalens. The corresponding phase profile *φ*(x, y) of the metalens follows the equal optical path principle^6,16^:

1 $$\varphi (x,y)=2\pi /\lambda \cdot (\sqrt{{x}^{2}+{y}^{2}+{f}^{2}}-f)$$

where *λ* is the incidence wavelength, *x* and *y* are the coordinates of the nanoblocks, and *f* is the designed focal length. The required phase is imparted based on the P-B phase via rotating the nanoblock with an angle *θ*(*x*, *y*) = *φ*(*x*, *y*)/2. Hence, each nanoblocks at coordinate (x, y) should be rotated with an angle

2 $$\theta (x,y)=\pi /\lambda \cdot (\sqrt{{x}^{2}+{y}^{2}+{f}^{2}}-f)$$

## Discussion

### Reflective metalens with on-center focusing

**Figure 3:**
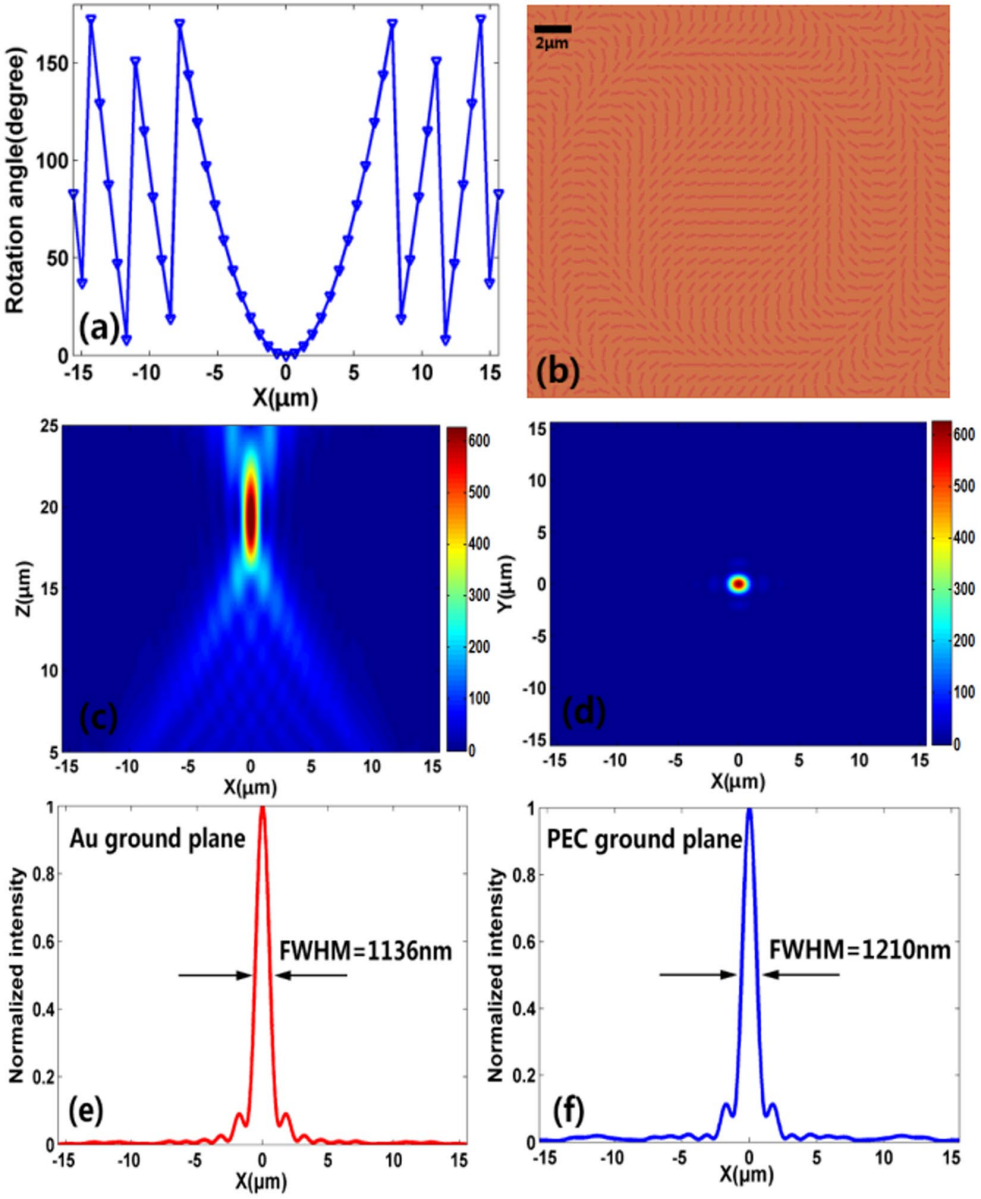
(**a**) Rotation angle of the nanoblocks along *x* axis for on-center focusing. (**b**) Top view of the center of the reflective metalens. The required phase is imparted by rotating the nanoblock with different angle *θ*(*x*, *y*). (**c** and **d**) Simulated intensity (|*E*|^2^) profile of the reflected light in *x-z* plane at *y* = 0 and in *x-y* plane at *z* = 20 μm with RCP incident light. (**e**) and (**f**) Vertical cuts of focal spot for the metalens with Au ground plane and PEC ground plane.

As illustrated in Fig. 3, the reflective metalens is designed to realize on-center focusing for RCP normal incident light at the wavelength of 1550 nm. The focal length is designed to be *f* = 20 μm, whereas the concept is scalable to any values. From Eq. 2, the rotation angle of the nanoblocks on the *x* axis is plotted in Fig. 3a. Top view of the reflective metalens is shown in Fig. 3b, where the targeting phase in Fig. 3a is imparted by rotating the nanoblock with a proper angle. Figure 3c,d show the simulated focal spot intensity (|*E*|^2^) profile at *x-y* and *x-z* planes, respectively. The plane metalens provides a strong focusing capability with a *N.A*. of ~0.65 and a focusing efficiency up to 85%. The focusing efficiency is defined as the fraction of the incident light that pass through a radius equal to three times of the FWHM spot size^25^.

### Sub-diffraction-limited characteristic of the focusing spot

The corresponding vertical cut of the focal spot is depicted in Fig. 3e. The full width at half maximum (FWHM) of the focal spot is 1136 nm (less than *λ/*2 *N.A*.), which indicates a sub-diffraction-limited focusing of our reflective metalens^27^. This effect can be attributed to our elaborately designed metalens structure. The gold ground plane is introduced to enhance the evanescent waves by the excitation of the surface plasmon. The nanoblocks arrays function as a coupler, which will convert the enhanced evanescent components into propagating waves^30^. With these two conditions, a metalens with sub-diffraction-limited focusing in the far-filed can be achieved. To verify the evanescent-filed enhancement mechanism of our designed metalens, the gold ground plane is replaced by a perfect electrical conductivity (PEC) ground plane. Without the enhanced evanescent waves by the excitation of the surface plasmon, the corresponding FWHM increased to 1210 nm (larger than *λ/*2 *N.A*.), suggesting the diffraction-limited characteristic of the focusing spot.

**Figure 4:**
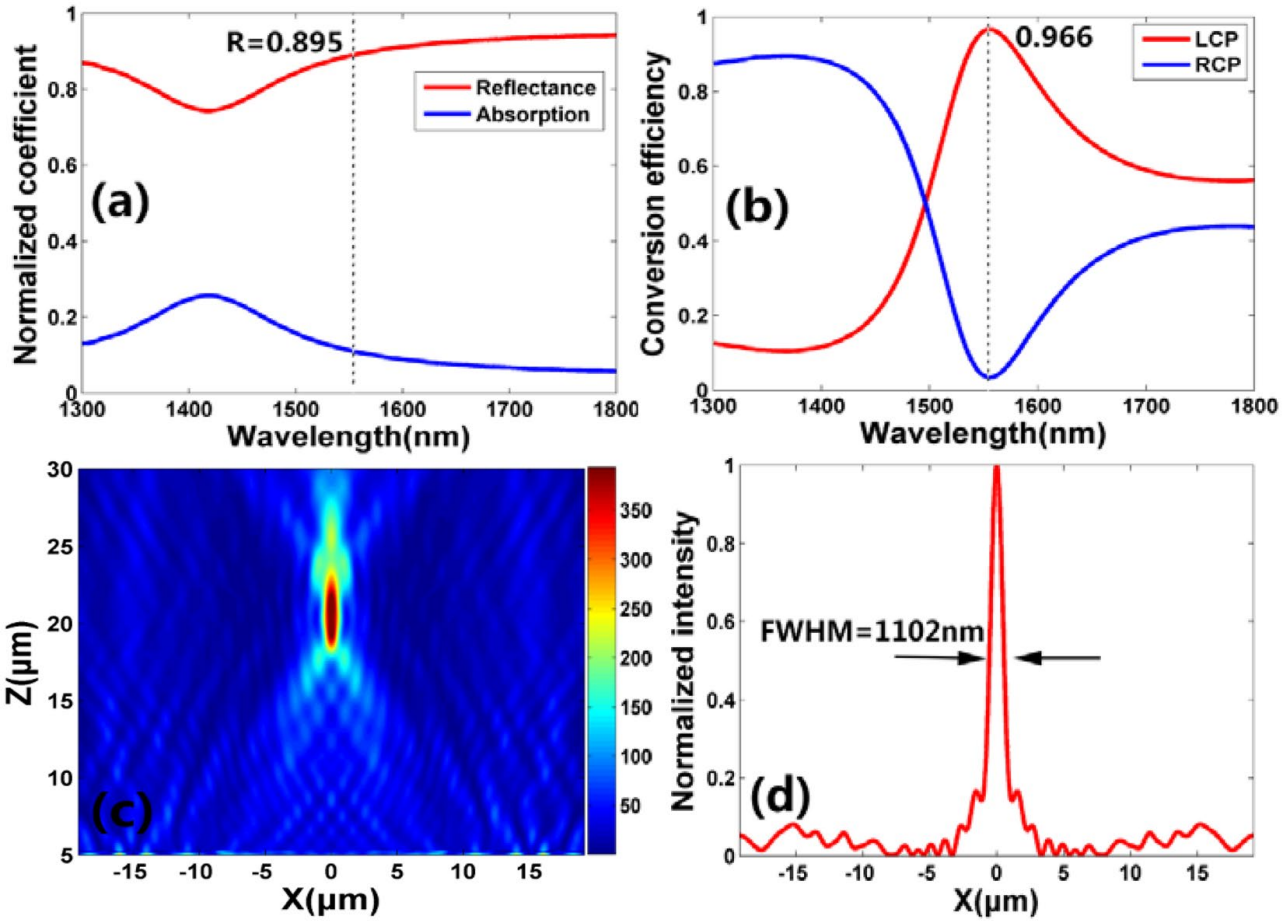
(**a**) Simulated reflection and absorption coefficient of the unit cell as a function of the incident wavelength. (**b**) Simulated polarization conversion efficiency of the unit cell as a function of the incident wavelength. The red and blue lines represent the proportion of the LCP and RCP in the total reflected power, respectively. (**c**) Simulated intensity (|*E*|^2^) profile of the reflected light in *x-z* plane at *y* = 0 with RCP incident light. (**d**) Vertical cut of focal spot for the metalens.

The metal-dielectric-metal (MIM) configuration generally also have high-efficiency for wavefront control^36^. To exhibit the advantage of our designed dielectric-dielectric-metal (DDM) configuration, a MIM configuration for focusing at the designed wavelength (1550 nm) is also taken into consideration. The Si nanoblock is replaced by Au nanoblock with the other configuration the same as Fig. 1. The detail optimized parameters are *h* = 430 nm, *h*_1_ = 200 nm, *h*_2_ = 150 nm, *W* = 180 nm, *L* = 700 nm and lattice constant *P* = 800 nm. Figure 4a,b show the reflectance and polarization conversion efficiency of the nanoblock as a function of the incidence wavelength, respectively. It can be observed that the reflectance is 89.5% and the polarization conversion efficiency is 96.6% at the designed wavelength (*λ* = 1550 nm). According to the definition in ref.^27^. which is defined as the ratio of the reflected power with opposite helicity to the total incident power, the polarization conversion efficiency should be 86.5%. The reflective metalens constructed of the Au nanoblocks is also demonstrated to realize on-center focusing. Figure 4c shows the simulated focal spot intensity (|*E*|^2^) profile at *x-z* plane. It is obvious that a focusing spot is exhibited at the designed focal length (*f* = 20 μm). The *N.A*. is ~0.69 and the simulated focusing efficiency is 73% at the designed wavelength. The corresponding vertical cut of the focal spot is shown in Fig. 4d, in which the FWHM of the focal spot is 1102 nm (less than *λ/*2 *N.A*.), indicating that the reflective metalens constructed of the Au nanoblocks also overcomes the diffraction limit. Therefore, despite reflective metalens with MIM configuration can also achieve diffraction-limited focusing, the focusing efficiency is less than that with DDM configuration.

**Figure 5:**
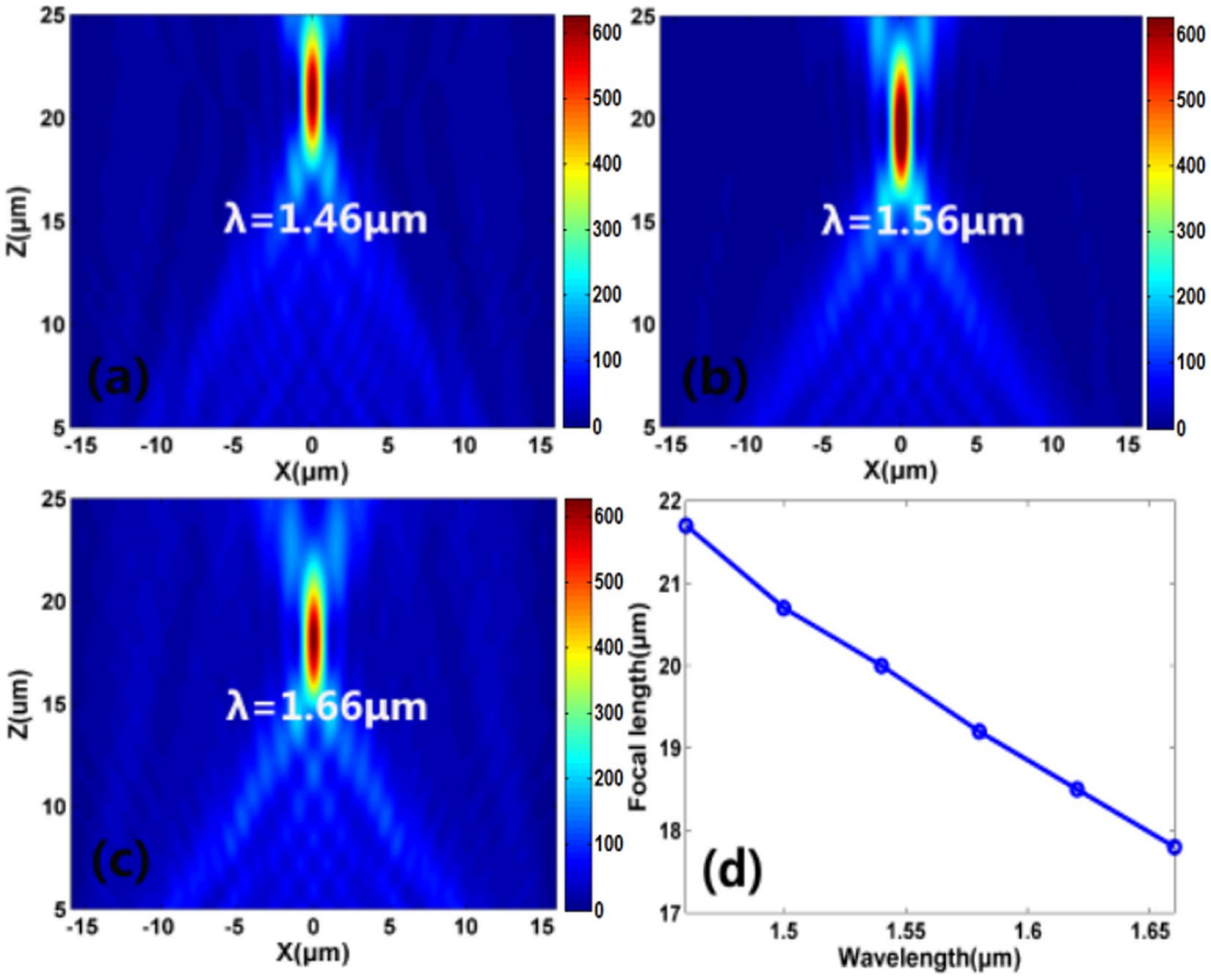
(**a**–**c**) Simulated intensity profiles in x-z plane for the metalens at wavelengths *λ* = 1.46 μm, 1.56 μm, 1.66 μm, respectively. (**d**) Simulated focal length as a function of the incident wavelength. The incident light is RCP that vertically illuminate on the metalens.

As mentioned above, by introducing the P-B phase, the designed nanoblock owns broadband characteristic (can achieve a phase coverage of 2*π* among a broad wavelength range). Hence, the metalens that constructed by the nanoblocks is bound to exhibit broadband focusing effect. Figure 5a–c show the simulated focal spot intensity profile at *x-z* plane for wavelengths *λ* = 1.46 μm, 1.56 μm, 1.66 μm, respectively. The focal length as a function of the incident wavelength is shown in Fig. 5d. From Fig. 5a–d, it can be seen that the designed reflective metalens exhibits focusing effect within a broad bandwidth and the focal length decreases as the increment of the wavelength. These results will provide helpful guidelines in modulating the focal length.

### Reflective metalens with off-center focusing

**Figure 6:**
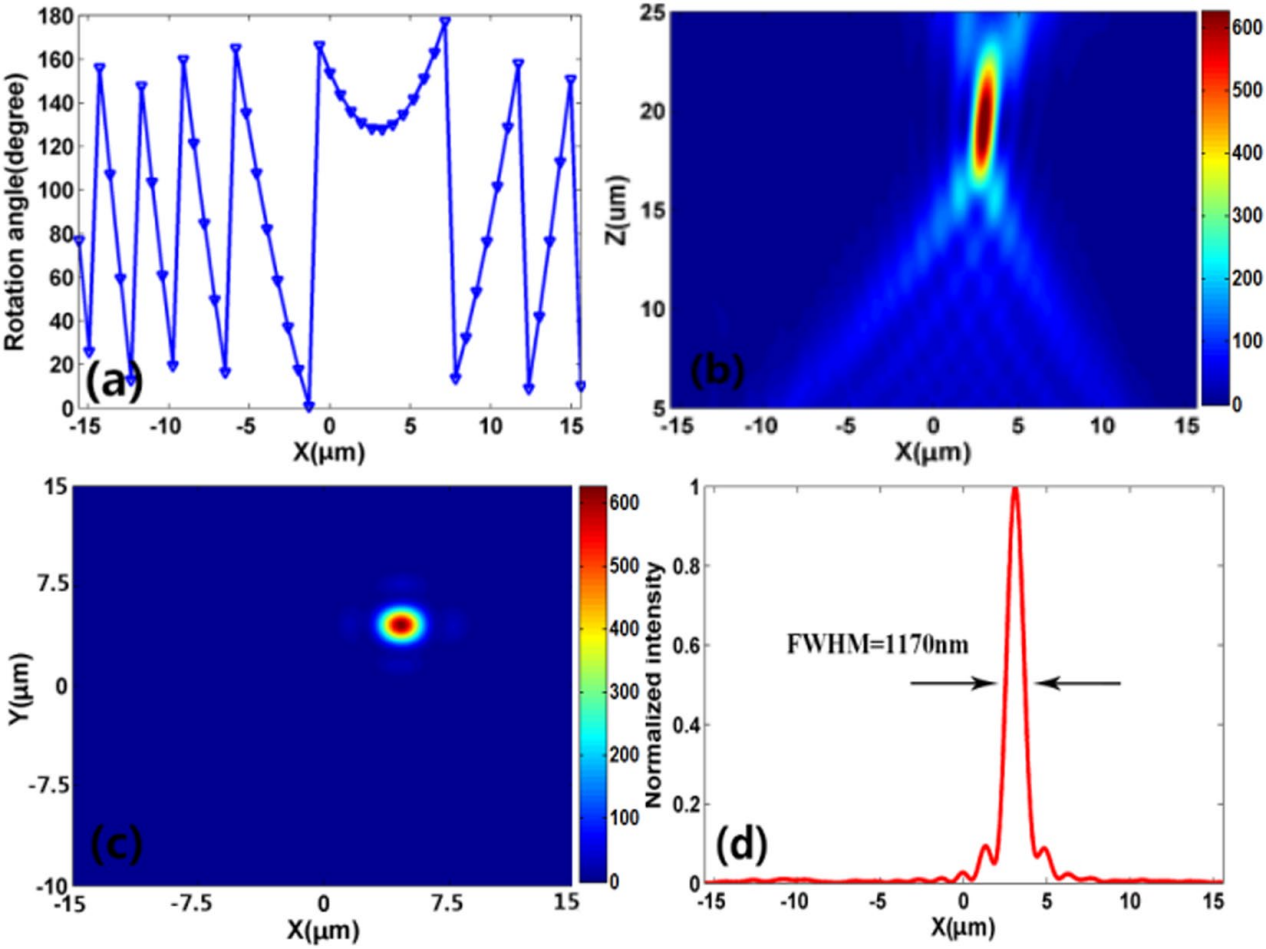
(**a**) Rotation angle of the nanoblocks along *x* axis for off-center focusing. (**b**) Simulated intensity (|*E*|^2^) profile of the reflected light in *x-z* plane at *y* = 0. (**c**) Simulated intensity profile of the reflected light in *x-y* plane at *z* = 20 μm. (**d**) Corresponding vertical cut of the metalens’ focal spot.

To focus the incident light to an arbitrary position A(*x*_1_, *y*_1_, *f*), each nanoblocks at coordinate (*x*, *y*) should be rotated with an angle

3 $$\{\begin{array}{c}\theta (x,y)=\pi /\lambda \cdot (\sqrt{{(x-{x}_{1})}^{2}+{(y-{y}_{1})}^{2}+{f}^{2}}-{f}_{1})\\ {f}_{1}=\sqrt{{{x}_{1}}^{2}+{{y}_{1}}^{2}+{f}^{2}}\end{array}$$

where *f*_1_ is the focal length, which defined as the distance from the focal point to the center of the nanoblocks plane. Based on Eq. 3, the rotation angle of the nanoblocks along the *x* axis are plotted in Fig. 6a. The off-center focusing spot is set at the location (3 μm, 3 μm, 20 μm). Figure 6b,c show the simulated focal spot intensity profile at *x*-*y* and *x*-*z* planes, respectively. The focal spot shows a slight shift from the expected location (3 μm, 3 μm, 20 μm), which results from imperfect phase change imparted by rotation the nanoblock. Besides, the simulated focusing efficiency is 85% at the designed wavelength. The corresponding vertical cut of the focal spot is shown in Fig. 6d, where the FWHM of the focal spot is 1170 nm (less than *λ/*2 *N.A*.), indicating that the off-center focusing is also overcomes the diffraction limit. Therefore, based on such a design principle, sub-diffraction-limited focusing at arbitrary position can be achieved, which will largely broaden its practical applications in laser-based microscopy, imaging and spectroscopy.

### Reflective metalens with dual spots focusing

**Figure 7:**
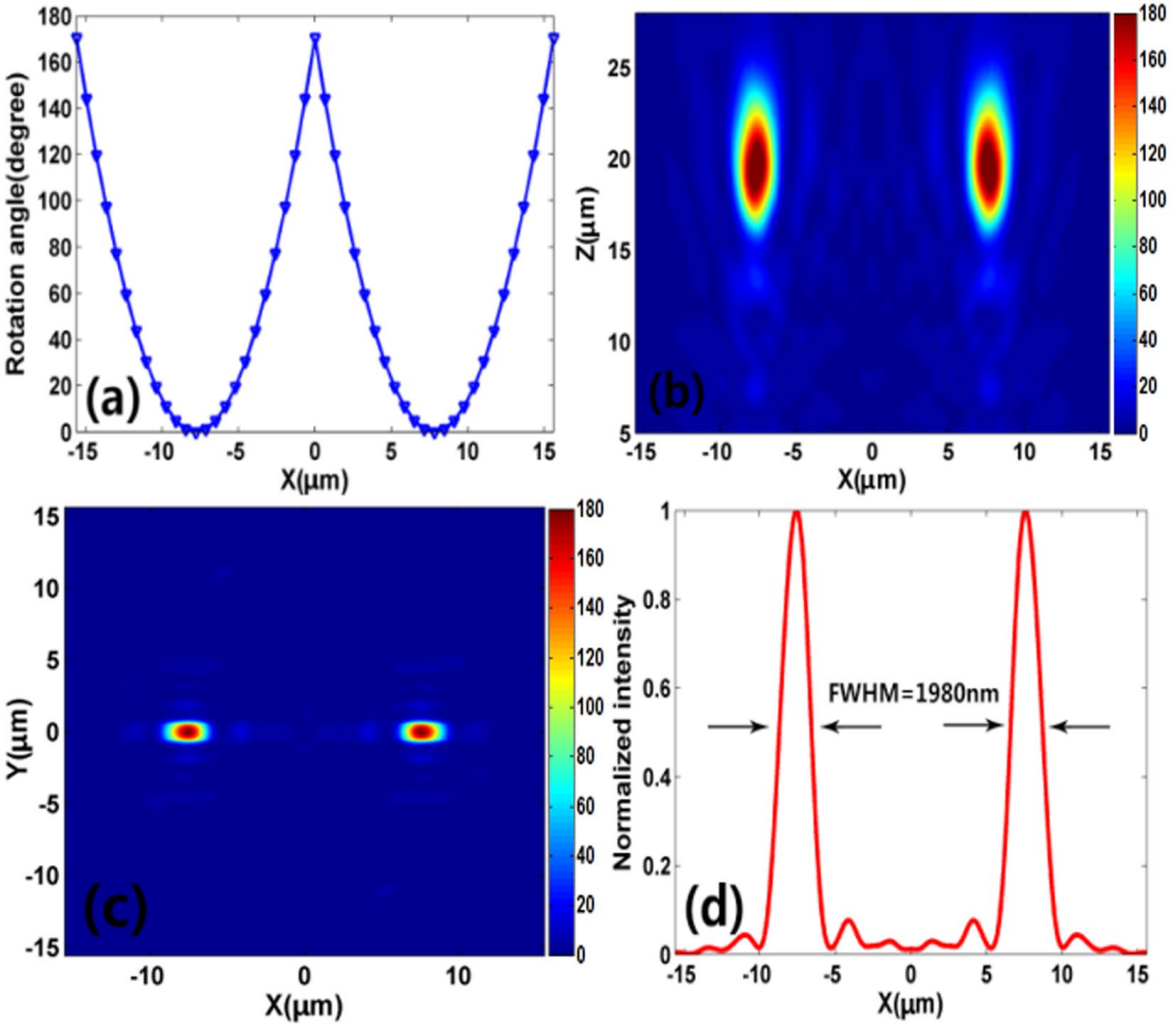
(**a**) Rotation angle of the nanoblocks along *x* axis for dual spots focusing at the horizontal direction. (**b**) Simulated intensity profile of the reflected light in *x-z* plane at *y* = 0. (**c**) Simulated intensity profile of the reflected light in *x-y* plane at *z* = 20 μm. (**d**) Corresponding vertical cut of the metalens’ focal spot.

The reflective metalens with dual spots focusing at the horizontal direction is demonstrated. The required rotation angle of the nanoblocks at coordinate (x, y) is expressed as

4 $$\theta (x,y)=\{\begin{array}{c}\pi /\lambda \cdot (\sqrt{{(x+{x}_{1})}^{2}+{y}^{2}+{f}^{2}}-f),x\le 0\\ \pi /\lambda \cdot (\sqrt{{(x-{x}_{1})}^{2}+{y}^{2}+{f}^{2}}-f),x > 0\end{array}$$

where ±*x*_1_ represents the locations of the dual spots. In this case, the two focal spots are located at (−8.4 μm, 0 μm, 20 μm) and (8.4 μm, 0 μm, 20 μm), respectively. The rotation angle of the nanoblocks along the x axis is shown in Fig. 7a, where the curve exhibits dual parabolic shape. Figure 7b,c show the simulated focal spot intensity profile at *x-y* and *x-z* planes, respectively. It can be observed that the focal spots occur at the expected locations. The simulated results also indicate that the two focal spots own equal focusing efficiency (42%) and the *N.A*. decreased to 0.38. Moreover, the FWHM of the two focal spots is shown in Fig. 7d. It can be observed that the FWHM of the focal spot increases to 1980 nm (less than *λ/*2 *N.A*.), indicating the sub-diffraction-limited characteristic of the focusing spot. Hence, dual spots sub-diffraction-limited focusing can be realized at the horizontal direction and such a design principle can be further applied to realize multi-spots focusing.

**Figure 8:**
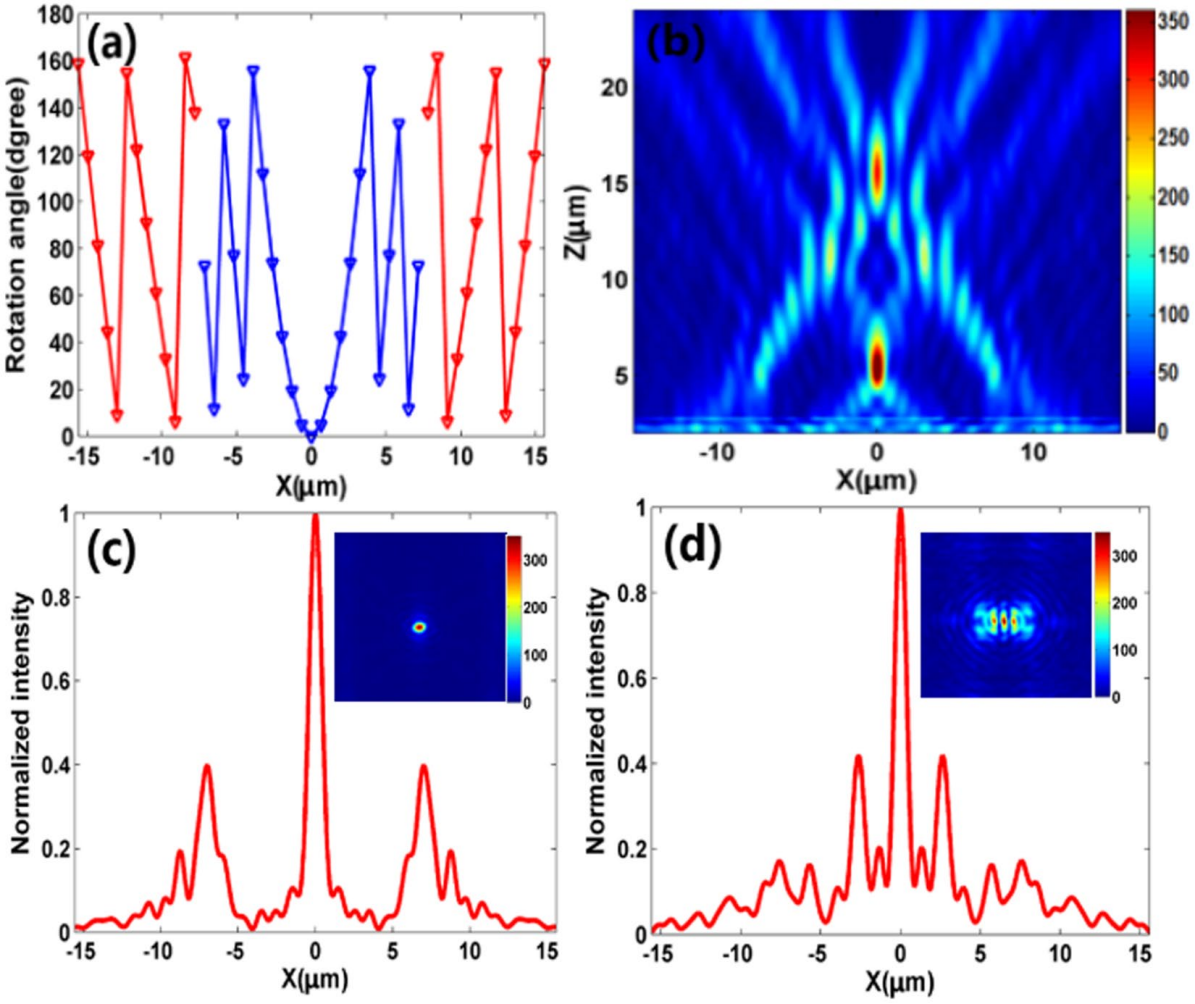
(**a**) Rotation angle of the nanoblocks along *x* axis for dual spots focusing at the longitudinal direction. The blue and red triangle lines represent the rotation angle for the focal spot *f*_*1*_ = 5 μm and *f*_*2*_ = 15 μm, respectively. (**b**) Simulated intensity profile of the reflected light in *x-z* plane at *y* = 0. (**c**) Corresponding vertical cut of the metalens’ focal spot (*f* = 5 μm). The inset is the simulated intensity profile of the reflected light in *x-y* plane at *z* = 5 μm. (**d**) Corresponding vertical cutting slice of the metalens’ focal spot (*f* = 15 μm). The inset is the simulated intensity profile of the reflected light in *x-y* plane at *z* = 5 μm.

To further explore the functionality of the reflective metalens, dual spots focusing at the longitudinal direction is demonstrated as well. Similarly, the required rotation angle of the nanoblocks at coordinate (*x*,*y*) should be expressed as

5 $$\theta (x,y)=\{\begin{array}{c}\pi /\lambda \cdot (\sqrt{{x}^{2}+{y}^{2}+{{f}_{1}}^{2}}-{f}_{1}),-x/4\le x\le x/4\\ \pi /\lambda \cdot (\sqrt{{x}^{2}+{y}^{2}+{{f}_{2}}^{2}}-{f}_{2}),x > x/4\cup x < -x/4\end{array}$$

where *f*_1_ = 5 μm and *f*_2_ = 15 μm are the designed focal lengths. Utilizing Eq. (5), the rotation angle of the nanoblocks along the *x* axis are depicted in Fig. 8a. The blue and red triangle lines represent the rotation angle for the focal spot *f*_1_ = 5 μm and *f*_2_ = 15 μm, respectively. The simulated focal spot intensity profile at *x-y* plane is shown in Fig. 8b, from which it can be observed that there are two focal spots locating at the designed positions along the longitudinal direction. The corresponding vertical cuts of the two focal spots are shown in Fig. 8c,d, respectively. The simulation results indicate that the focusing efficiency at focal length *f*_1_ = 5 μm and *f*_2_ = 15 μm are 34% and 36%, respectively. This vision-violated results are mainly caused by the fact that some of reflected lights pass through the two focal spots, resulting in a higher focusing efficiency for the top focal spot.

### Reflective metalens with different *N.A*

**Figure 9:**
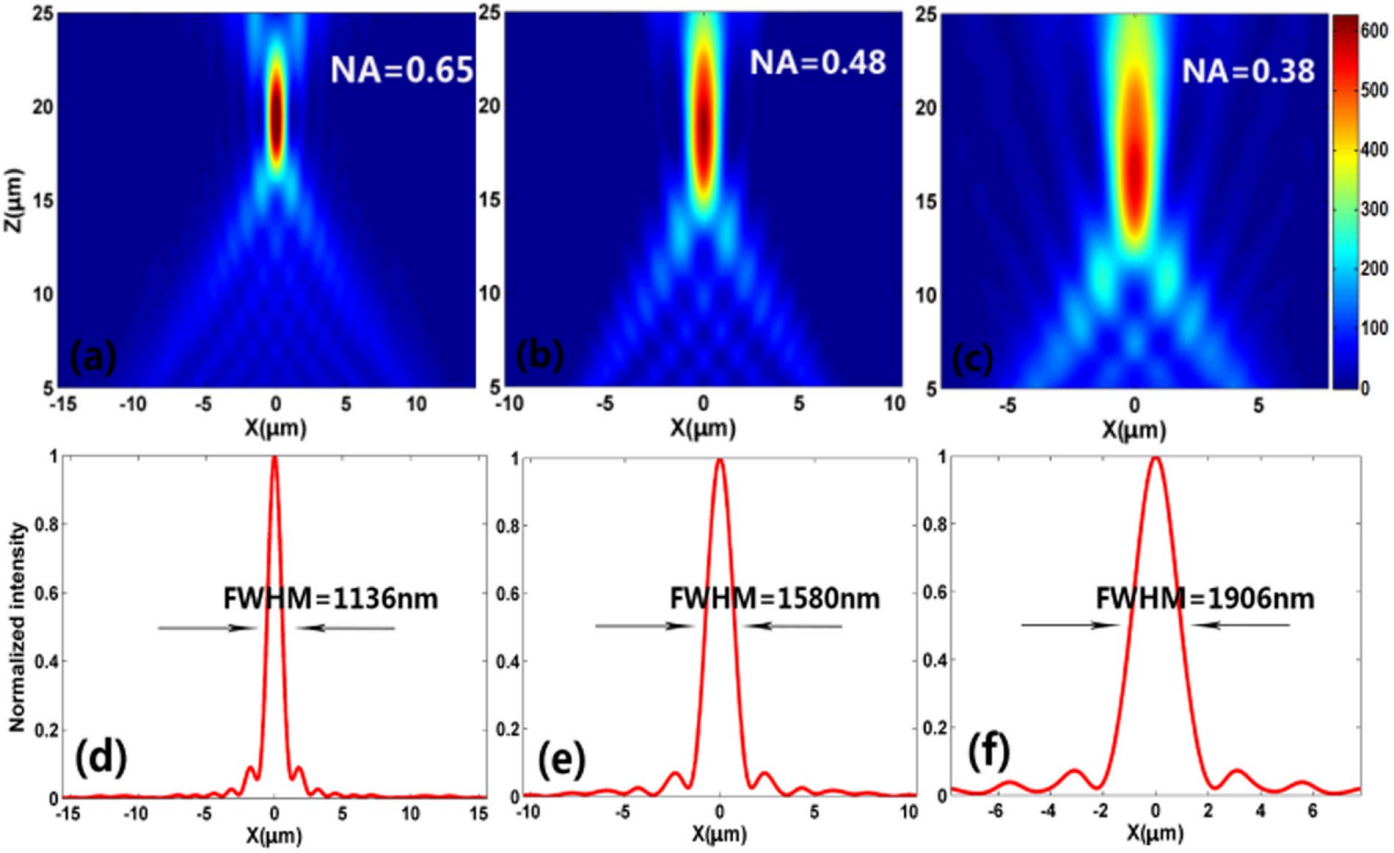
(**a**–**c**) Simulated intensity profile of the reflected light in *x-z* plane at *y* = 0 for the metalenses with different N.A. The focal length of the three metalenses is *f* = 20 μm. (**d**–**f**) Corresponding vertical cuts of the metalenses’ focal spots.

As we known, *N.A*. is regarded as one of the most critical parameters to characterize the metalens. Here, *N.A*. is defined as *N.A*. = *sin[tan* ^*−*1^ *(D/*2* f)*], where *f* is the focal length and *D* is the width of the metalens. Figure 9a–c show the simulated focal spot intensity profile at *x-z* plane for three metalens with different *N.A*., but with the same focal length (*f* = 20 μm). The FWHM of the focal spots for the three metalenses are shown in Fig. 9d–f. It can be observed that for a fixed focal length, the *N.A*. decreased as the reducing of the size of the metelens. Besides, we can confirm that it will show better focusing property (narrow FWHM and high focusing efficiency) for the metalenses with larger *N.A*. For our demonstrated on/off-center focusing (*f* = 20 μm), the calculated *N.A*. is only 0.65, which can be ascribed to the restriction of the simulated region caused by the computing ability in our group. According to its definition, the *N.A*. will increase with the decreasing of the focal length for a fixed width *D*. As for our demonstrated dual spots focusing at the longitudinal direction, the calculated *N.A*. can reach 0.84 for the lower focal spot (*f* = 5 μm), which is larger than most reported metalens^16,27,28^. These results will provide helpful guidelines in designing metalens with high focusing properties.

In summary, an ultra-thin planar reflective metalens with sub-diffraction-limited and multifunctional focusing has been investigated. Based on the principle of equal optical path, the formulas of the requiring phase distributions for multifunction focusing are derived in detail. Following the formulas, on-center, off-center and dual spots focusing (at horizontal and longitudinal directions) are demonstrated. It worth noting that all these focusings are sub-diffraction-limited due to the evanescent-filed enhancement mechanism in our designed metalens. Moreover, the *N.A*. dependence on the area of the metalens is discussed. With such a design principle in our reflective metalens, one can obtain the sub-diffraction-limited focusing at any positions with high focusing properties. These results will provide helpful guidelines in designing super-resolution light imaging and sensing systems.

## Methods

### Simulations

The performance of the the proposed metalenses are characterized by using the three-dimensional finite difference time domain (FDTD) method from Lumerical Inc. For the simulation of the unit cell, periodic boundary conditions are applied along the *x* and *y* axis and perfectly matched layers(PML) is applied along the *z* axis. For the simulation of the metalenses, PML are applied along the three axis for the specific phase elements of the designed metalens. The simulated total area of the metalens is 33.8 × 33.8 μm^2^ with 53 × 53 unit cells.
